# Differences in quadriceps but not hamstrings strength in runners with varying durations of patellofemoral pain

**DOI:** 10.3389/fspor.2025.1612257

**Published:** 2025-10-09

**Authors:** Mianchuan He, Huijuan Shi, Shengxing Fu, Hui Liu

**Affiliations:** ^1^ Biomechanics Laboratory, College of Human Movement Science, Beijing Sport University, Beijing, China; ^2^ Key Laboratory for Performance Training & Recovery of General Administration of Sport, Beijing, China; ^3^ China Institute of Sport and Health Science, Beijing Sport University, Beijing, China

**Keywords:** patellofemoral pain, running, knee, isokinetic torque, symmetry

## Abstract

**Background:**

Patellofemoral pain (PFP) is a common injury among runners. The quadriceps and hamstrings strength in patients with different durations of PFP is still unclear. The purpose of this study is to evaluate the isokinetic strength characteristics of quadriceps and hamstrings and the symmetry of muscle strength in runners at different stages of PFP, providing a theoretical basis for the prevention and rehabilitation of PFP.

**Methods:**

Ten patients with a short PFP duration (short-term group, <3 months), eleven patients with a long PFP duration (long-term group, >12 months), and sixteen healthy runners (control group) participated. Bilateral quadriceps and hamstrings isokinetic strength data were collected from all individuals. A one-way ANOVA was performed to evaluate the effect of disease duration on knee joint isokinetic strength and the strength symmetry index.

**Results:**

The short-term group had significantly lower peak quadriceps torque than the long-term (*P* = 0.009) and control groups (*P* = 0.015). However, peak quadriceps torque did not significantly differ between the long-term and control groups (*P* = 0.639). The strength symmetry index of the quadriceps (*P* = 0.250) and hamstrings (*P* = 0.541), as well as the peak torque of the hamstrings (*P* = 0.087), did not differ significantly across the three groups. No significant differences in the H/Q ratio among the short-term group, the long-term group, and the control group (*P* = 0.440).

**Conclusion:**

Quadriceps strength varied across stages of PFP, with individuals in the short-term group showing weaker strength than the healthy, while those in the long-term group exhibited strength comparable to the healthy. Runners with different durations of PFP demonstrate little difference in hamstring strength, H/Q ratio, and muscle symmetry from healthy.

## 1 Introduction

Patellofemoral Pain (PFP) is one of the most common chronic lower limb injuries. It mainly manifests as anterior knee pain during activities such as running, squatting, and jumping (1). PFP is very common in physically active populations, especially runners, with an annual incidence of approximately 4% to 21% (2). The treatment effect for PFP is often unsatisfactory (3). After the initial onset of PFP symptoms, 70%–90% of patients may experience recurrence or chronic symptoms (4). Studies have shown that more than half of patients with PFP experience pain that lasts for several years (3). Although rest or reducing training intensity can alleviate pain to some extent (5), many PFP runners still run intermittently, which may exacerbate the symptoms. The chronic nature of PFP may lead to patellofemoral osteoarthritis (6), which can affect runners' athletic lives and quality of life.

The insufficiency and imbalance of muscle strength around the knee joint are closely related to the occurrence and persistence of PFP. Quadriceps strength is an important factor affecting dynamic patellar stability (5). Some studies suggest that weak quadriceps strength is a significant risk factor for PFP (7, 8). However, other studies have found that greater quadriceps strength is also associated with the occurrence of PFP (9). The occurrence of PFP may also be related to an imbalance in muscle strength around the knee joint (10). The hamstrings-to-quadriceps strength ratio (H/Q ratio) is a clinical measure widely used to assess lower extremity muscle balance and has been identified as a critical component of an athlete's readiness to return to sport after an injury (11). A low H/Q ratio has also been associated with an increased risk of an anterior cruciate ligament (ACL) injury (12). However, the relationship between the H/Q ratio and PFP remains unclear. Additionally, asymmetry in lower limb strength is also considered a cause of many injuries (13). A difference of more than 20% in bilateral lower limb strength is associated with an increased risk of injury in athletes (14). However, whether bilateral lower limb strength is symmetrical in patients with PFP remains controversial. Some studies have demonstrated asymmetrical lower limb strength in patients with PFP (15), whereas others have reported that the strength of the injured lower limb in patients with PFP is relatively symmetrical to that of the uninjured contralateral lower limb (16). Therefore, further research is needed to verify these findings.

The quadriceps and hamstrings strength in patients with different durations of PFP may exhibit varying characteristics. Analyzing these differences can help doctors develop personalized rehabilitation programs, thereby improving treatment efficacy. Current research on PFP with different durations mainly focuses on gait indicators (17, 18), while the patterns of muscle strength changes remain unclear. Previous research has found significant differences in gait characteristics between patients with chronic and acute PFP (18), which may be related to variations in muscle strength. Therefore, identifying the characteristics of quadriceps and hamstrings muscle strength in patients with PFP at different stages could help develop more targeted treatment strategies.

This study aimed to evaluate the isokinetic strength of the bilateral quadriceps and hamstrings, as well as the muscle strength symmetry index in different durations of PFP. The results will provide a reference for developing treatment strategies for patients with patellofemoral pain. The hypotheses of this study were: (1). The quadriceps in healthy individuals would be stronger than those in the short-term group, and patients in the short-term group would be stronger than those in the long-term group. (2) The H/Q ratio of patients in the short-term group would be higher than that in healthy individuals, and patients in the long-term group would be higher than those in the short-term group. (3) The muscle strength symmetry index of the quadriceps and hamstrings in the short-term group would be lower than that in healthy individuals, and patients in the long-term group would be lower than those in the short-term group.

## 2 Materials and methods

### 2.1 Participants

This study adopted a cross-sectional research design and recruited participants from an amateur runner population aged 18–40 years (Table 1). An apriori power analysis was conducted using G*Power, version 3.1.9.2 (University Kiel, Germany) to determine the minimum sample size. The effect size was estimated based on the quadriceps peak torque data from a cross-sectional study of patellofemoral pain (31). The final calculation yields a minimum sample size of 10 individuals per group (effect size = 0.6, α = 0.05, β = 0.20). A total of 10 patients who had experienced PFP symptoms within the last 3 months (short-term group, six males and four females), 11 patients with PFP symptoms persisting for more than 12 months (long-term group, seven males and four females), and 16 healthy runners (control group, nine males and seven females) were ultimately recruited. The isokinetic muscle strength of the quadriceps and hamstrings of the participants in each group was collected and analyzed, and then compare the differences between the groups. All runners with PFP continued running during their illness without any treatment intervention, and there were no significant differences in sex, age, or weekly running distance among the three groups.

**Table 1:** Demographic characteristics of participants.

Variables	Control	Short-term	Long-term	*P*-value
Participants	16	10	11	–
Age (years)	22 ± 2	23 ± 4	22 ± 2	.662
BMI	21.58 ± 1.95	22.04 ± 2.44	21.21 ± 1.75	.631
Duration of Symptoms (months)	/	1.9 ± 1.6	34.9 ± 22.4	.000
Weekly Running Distance (km)	16.31 ± 9.67	19.50 ± 15.06	22.18 ± 22.53	.696

The diagnostic and screening criteria for PFP were based on the *Patellofemoral Pain: Clinical Practice Guidelines Linked to the International Classification of Functioning, Disability and Health* published by the Academy of Orthopaedic Physical Therapy of the American Physical Therapy Association (19). The inclusion criteria were: (1) patellofemoral joint pain during or after activities such as running, jumping, ascending/descending stairs, or single-leg squatting; (2) a running history of at least 2 years with a weekly mileage of no less than 10 kilometers. The exclusion criteria were: (1) a history of patellar dislocation, subluxation, or osteoarthritis of the knee joint; (2) a history of lower limb orthopedic surgery; (3) any other neurological, musculoskeletal, or cardiovascular diseases. The control group required participants to have no symptoms of knee pain, no history of lower limb injury or surgery in the past year, and no limitations in knee joint mobility. All participant screenings were conducted by an experienced rehabilitation therapist, and all participants signed an informed consent form approved by the university institutional review board before participating in the experiment (ref No. 2022045H).

### 2.2 Procedures

**Figure 1:**
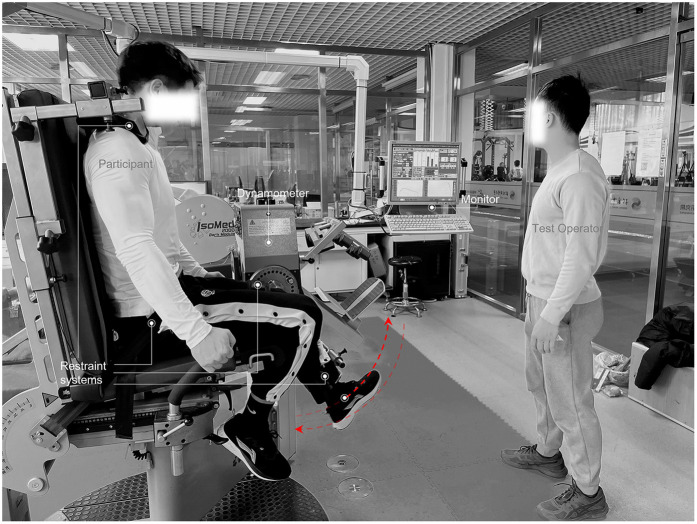
Strength testing in isometric knee extension and flexion.

Before the test, each participant completed a standardized 10 min general warm-up. An IsoMed2000 isokinetic dynamometer (D&R Ferstl GmbH, Hemau, Germany) was used to measure the concentric peak torque of the quadriceps and hamstrings at an angular velocity of 60°/s. The quadriceps range of motion was 90° to 10° of knee flexion, and the hamstrings range was 10° to 90° of knee flexion. Participants were seated with their backs against the backrest, which was set at a 75° angle (0° = full extension). The knee's rotational axis was aligned with the dynamometer's mechanical axis using a laser pointer, with the lateral femoral epicondyle representing a bony reference point. The trunk, pelvis, and distal femur were secured with wide straps (Figure 1). The strength of the knee flexor and extensor muscles on the injured and uninjured limb was evaluated concentrically at a speed of 60°/s. Before testing, three repetitions of knee flexion-extension movements were performed as a warm-up to familiarize and understand the test. This was followed by three sets of maximal effort concentric knee extensions and flexions, with each set consisting of five repetitions and a one-minute rest interval between sets. The testing sequence strictly followed the order of the uninjured leg first followed by the injured leg. In the control group, the dominant leg was tested first, defined as the leg used to kick a ball, followed by the non-dominant leg. Pain was monitored using the Visual Analog Scale (VAS) to ensure that participants were tested within their pain-tolerable range.

The study indicators include peak torque per body weight (Nm/kg), angle of peak torque, hamstrings-to-quadriceps ratio, total work (J/kg), and muscle strength symmetry index (%). The angle of peak torque refers to the knee joint angle at which the peak torque occurs, reflecting the optimal force application angle and guiding targeted rehabilitation programs (20). The hamstrings-to-quadriceps strength ratio (H/Q ratio) was calculated using the peak torque values of the knee flexors and extensors (21). Total work represents the total energy produced by all the muscles involved in the movement, calculated as the area under the torque-angle curve for a single movement, and was normalized to body weight. Total work comprehensively reflects the energy release capability of all muscle fibers involved in the movement and is a reliable measure of muscle function. The muscle strength symmetry index was calculated as follows: injured limb value/ uninjured limb value × 100% (22). In the control group, the muscle strength symmetry index was calculated as follows: non-dominant limb value/dominant limb value × 100.

### 2.3 Statistical analysis

One-way ANOVA was used to compare the differences in the selected indicators among the three groups of participants. The dominant leg of the control group was chosen for comparative analysis with the injured leg of PFP patients (40). All statistical analyses were performed using SPSS version 26.0 (SPSS, Chicago, IL, USA). A type I error rate ≤0.05 was chosen as an indication of overall statistical significance in ANOVA. In the *post-hoc* comparisons following the ANOVA, the Type I error rate was adjusted to 0.017 to ensure that the overall Type I error rate did not exceed 0.05.

## 3 Results

One-way ANOVA showed that there were no significant differences in the quadriceps peak torque (*P* = 0.017) and total work of knee extension (*P* = 0.035) among the short-term group, the long-term group, and the control group (Table 2). The quadriceps peak torque in the short-term group was significantly lower than that in the long-term group (*P* = 0.009) and the control group (*P* = 0.015), whereas there was no significant difference between the long-term and control groups (*P* = 0.639). The total work of knee extension in the short-term group was significantly lower than that in the long-term group (*P* = 0.015), with no significant difference between the control group and the short-term (*P* = 0.033) and long-term groups (*P* = 0.583) (Table 2). There were no significant differences among the three groups in terms of the peak torque of the hamstrings (*P* = 0.087), total work of knee flexion (*P* = 0.116), peak torque angle of knee flexion (*P* = 0.276), and knee extension (*P* = 0.257).

**Table 2:** Peak torque, peak torque angle, total work and H/Q ratio (M ± SD).

Indicator		Control	Short-term	Long-term	*P*-value
Hamstrings	Peak Torque (N·m/kg)	1.84 ± 0.27	1.58 ± 0.35	1.75 ± 0.30	.087
	Peak Torque Angle (°)	37.43 ± 4.80	35.37 ± 3.76	34.67 ± 4.91	.276
	Total Work (J/kg)	1.73 ± 0.30	1.49 ± 0.32	1.68 ± 0.21	.116
Quadriceps	Peak Torque (N·m/kg)	2.71 ± 0.32	2.30 ± 0.44^a^	2.78 ± 0.44^b^	.017
	Peak Torque Angle (°)	60.53 ± 3.04	61.67 ± 5.12	63.30 ± 4.18	.257
	Total Work（J/kg）	2.15 ± 0.29	1.85 ± 0.37	2.22 ± 0.34^b^	.035
H/Q Ratio		0.75 ± 0.07	0.76 ± 0.12	0.71 ± 0.09	.391

^a^ Indicates a significant difference compared with the control group^.^

^b^ Indicates a significant difference compared with the short-term group.

One-way ANOVA showed that there were no significant differences in the H/Q ratio among the short-term group, the long-term group, and the control group (*P* = 0.440, Table 2), and no significant differences in the symmetry index of the hamstrings (*P* = 0.541) and the quadriceps (*P* = 0.250) among the short-term group, the long-term group, and the control group (Table 3).

**Table 3:** The symmetry index of quadriceps and hamstrings muscle strength (M ± SD).

Muscle	Control (%)	Short-term (%)	Long-term (%)	*P*-value
Hamstrings	99.84 ± 10.10	95.63 ± 9.74	99.25 ± 9.02	.250
Quadriceps	95.32 ± 9.16	92.31 ± 6.15	98.53 ± 8.90	.541

## 4 Discussion

This study found significantly lower peak isokinetic torque of the quadriceps in patients with PFP symptoms lasting less than 3 months than in the control group. This result is consistent with previous studies, which have shown significantly lower isokinetic strength of the quadriceps in patients with PFP than in healthy individuals, with a difference ranging from 20% to 60% (23–25). Quadriceps atrophy might be the primary reason for the decreased isokinetic strength, as studies have found significant physiological atrophy of the quadriceps in patients with PFP compared with healthy individuals (26). However, some studies found no significant correlation between the peak isokinetic torque of the quadriceps and its cross-sectional area in patients with PFP. This suggests that in addition to quadriceps atrophy, factors such as neuromuscular changes, biomechanical changes around the patellofemoral joint, and pain inhibition responses may also affect quadriceps function (15). Pain inhibition may be a critical factor in the decreased isokinetic strength of the quadriceps (27, 28). The theory suggests that muscles associated with pain are inhibited during activity, while the activity of the corresponding antagonist muscles is promoted, leading to reduced parameters of speed and force output (29). This study also found that patients with PFP symptoms lasting more than 12 months had significantly higher isokinetic strength of the quadriceps than those in the short-term group symptoms, and their strength was similar to that of the control group. Notably, the findings of this study contradict our initial hypothesis that individuals with short-term PFP would demonstrate greater quadriceps strength than those with long-term PFP. Instead, the long-term group exhibited significantly higher quadriceps strength. This inconsistency may be explained by pain adaptation and neuromuscular compensation mechanisms. While individuals with short-term PFP may experience acute pain inhibition that suppresses quadriceps activation, those with long-term PFP may develop coping strategies, such as altered motor control, postural adjustments, or desensitization to pain, which help restore quadriceps function over time. However, this interpretation is speculative, as the cross-sectional design of the study does not allow us to confirm recovery over time. Longitudinal studies are needed to directly track muscle strength progression in PFP patients. Hodge and Tucker found that during strength testing in isometric knee extension, local pain can redistribute neural activation within and between muscles (30). Although pain reduces the discharge frequency of original motor neurons, it also recruits more new motor units to participate in muscle activity. As the duration of pain increases, patients might develop new neuromuscular control strategies through continued exercise, changing their biomechanical behavior and movement patterns, and ultimately normalizing quadriceps strength (31). Future research could further explore the relationship between lower limb biomechanics and quadriceps strength in patients with different PFP durations.

This study found no significant differences in hamstring peak torque across groups. In contrast, Werner et al. observed reduced hamstring strength in PFP patients compared to healthy controls (25). One potential reason for this discrepancy may be the participant characteristics: Werner's study recruited individuals from the general population with no specific athletic background, whereas our study involved amateur runners with regular training habits. Specifically, the average weekly running distances in our sample ranged from 16.31 km to 22.18 km across groups, with no significant difference among them (*P* = 0.696). This suggests a relatively high and consistent activity level, which may have helped maintain hamstring strength despite the presence or duration of symptoms. Nonetheless, this remains a plausible but unconfirmed interpretation that requires further investigation with longitudinal designs.

The peak torque angle of isokinetic strength reflects the optimal joint angle for muscle exertion and has a certain reference value for formulating rehabilitation programs. This study found no significant difference in the isokinetic peak torque angle of the quadriceps and hamstrings among the short-term, long-term, and control groups. This result is consistent with those of Werner et al., who also did not find significant differences in the isokinetic peak torque angle of the quadriceps and hamstrings between patients with PFP and control groups (25). A possible reason is that the peak torque angle mainly depends on individual morphology and muscle physiological characteristics, which are less affected by PFP.

This study found that the total work of knee extension was lowest in the short-term group, significantly lower than that in the long-term group, but not significantly different from the healthy controls. Although the difference between the short-term group and healthy controls did not reach statistical significance, the data showed a lower mean value in the short-term group. This may reflect a type II error due to limited sample size or variability. In contrast, the long-term group showed total work comparable to healthy individuals, possibly indicating neuromuscular adaptation over time. These findings suggest a potential recovery pattern of muscle performance across PFP duration, which warrants further investigation in longitudinal studies.

This study found no significant differences in the H/Q ratio among the short-term, long-term, and healthy controls, consistent with previous research. For example, Brown et al. reported no significant H/Q ratio differences between male rugby players with PFP and healthy athletes (32). The H/Q ratio reflects the functional balance between the knee flexors and extensors, which is important for joint stability (33). However, it is influenced by multiple factors such as angular velocity, joint position, and task specificity (34, 35). In most studies, including ours, the ratio is calculated using peak torque values at a single joint angle, which may not capture imbalances that occur during functional movements or across different ranges of motion. This methodological limitation could contribute to the consistently non-significant findings in the literature.

This study did not find significant differences in quadriceps or hamstring strength symmetry index among the short-term, long-term, and control groups. All participants demonstrated symmetry indices above 90%, exceeding the commonly accepted threshold of 85%–90% used for return-to-sport decisions in ACL rehabilitation (36). This suggests that PFP, in both short-term and long-term stages, may not lead to substantial bilateral strength imbalance in active populations such as runners. These findings are consistent with those of Ouazzani et al., who reported no significant side-to-side strength differences in patients with PFP (16). However, this lack of asymmetry could reflect limitations in the sensitivity of current testing methods. Isokinetic measurements at a single angular velocity under controlled conditions may not detect subtle asymmetries that manifest during fatigue, dynamic movement, or sport-specific tasks. Moreover, it remains unclear whether strength symmetry is functionally meaningful in PFP, which often involves biomechanical and neuromuscular factors beyond pure force output. Future research should consider incorporating more dynamic and functional assessments to evaluate asymmetry in PFP more comprehensively.

Clinically, this study highlights the importance of considering symptom duration when designing rehabilitation strategies for PFP. In the short-term group, reduced quadriceps strength suggest that early rehabilitation should focus on improving quadriceps function and relieving pain-related inhibition. Strengthening exercises combined with basic neuromuscular training may help restore knee performance in these patients. In contrast, individuals with long-term PFP exhibited muscle strength and symmetry comparable to healthy controls, suggesting that basic strength capacity may not be the limiting factor in long-term PFP. For these patients, rehabilitation may require a broader focus on movement retraining, addressing kinetic chain deficits, psychological factors, or sport-specific demands.

This study has several limitations that should be acknowledged. First, its cross-sectional design limits the ability to infer causal relationships between the duration of PFP and muscle strength characteristics. Second, although the sample size was determined based on power analysis, it may still reduce statistical sensitivity to detect subtle differences between groups. Third, isokinetic strength testing was performed at a single angular velocity (60°/s) and included only concentric contractions, which may not fully capture performance differences under more functional or sport-specific conditions. Fourth, the sample consisted entirely of amateur runners, so generalizing these findings to other populations (e.g., elite athletes, sedentary individuals) should be done with caution, as muscle strength characteristics and PFP manifestations may differ across groups with distinct activity levels. Despite these limitations, the findings contribute to a better understanding of how muscle strength characteristics vary with the duration of PFP and may provide valuable guidance for more targeted rehabilitation strategies in clinical settings.

## 5 Conclusion

Individuals in the short-term group exhibited weaker quadriceps isokinetic strength than the control group, whereas those in the long-term group showed similar quadriceps isokinetic strength to that of the controls. These findings highlight the need to prioritize quadriceps strengthening in short-term PFP rehabilitation.

## Data Availability

The raw data supporting the conclusions of this article will be made available by the authors, without undue reservation.
